# Role of bZIP Transcription Factors in Response to NaCl Stress in *Tamarix ramosissima* under Exogenous Potassium (K^+^)

**DOI:** 10.3390/genes14122203

**Published:** 2023-12-13

**Authors:** Yahui Chen, Min Zhang, Dezong Sui, Jiang Jiang, Lei Wang

**Affiliations:** 1 Jiangsu Academy of Forestry, Nanjing 211153, China; chenyahui@njfu.edu.cn (Y.C.); nmzhang@163.com (M.Z.); suidezong@163.com (D.S.); 2 Collaborative Innovation Center of Sustainable Forestry in Southern China of Jiangsu Province, Nanjing Forestry University, Nanjing 210037, China

**Keywords:** bZIP transcription factors, NaCl stress, halophyte, transcriptome, NaCl toxicity

## Abstract

Salt stress is a significant environmental factor affecting plant growth and development, with NaCl stress being one of the most common types of salt stress. The halophyte, *Tamarix ramosissima* Ledeb (*T*. *ramosissima*), is frequently utilized for the afforestation of saline-alkali soils. Indeed, there has been limited research and reports by experts and scholars on the regulatory mechanisms of basic leucine zipper (*bZIP*) genes in *T*. *ramosissima* when treated with exogenous potassium (K^+^) to alleviate the effects of NaCl stress. This study focused on the *bZIP* genes in *T. ramosissima* roots under NaCl stress with additional KCl applied. We identified key candidate genes and metabolic pathways related to bZIP and validated them through quantitative real-time PCR (qRT-PCR). The results revealed that under NaCl stress with additional KCl applied treatments at 0 h, 48 h, and 168 h, based on Pfam protein domain prediction and physicochemical property analysis, we identified 20 related *bZIP* genes. Notably, four *bZIP* genes (*bZIP*_2, *bZIP*_6, *bZIP*_16, and *bZIP*_18) were labeled with the plant hormone signal transduction pathway, showing a predominant up-regulation in expression levels. The results suggest that these genes may mediate multiple physiological pathways under NaCl stress with additional KCl applied at 48 h and 168 h, enhancing signal transduction, reducing the accumulation of ROS, and decreasing oxidative damage, thereby enhancing the tolerance of *T*. *ramosissima* to NaCl stress. This study provides gene resources and a theoretical basis for further breeding of salt-tolerant *Tamarix* species and the involvement of bZIP transcription factors in mitigating NaCl toxicity.

## 1. Introduction

In the context of the global climate instability and deteriorating ecological environment, an increasing number of vegetation, including crops and trees, are subjected to multiple abiotic stresses such as drought, cold, and salinity [1,2,3]. Among these, salt stress is a primary factor affecting plant growth and development [4]. Under salt stress, plant roots become less vigorous, cell membrane permeability decreases, and antioxidant enzyme activities decline, all of which inhibit plant growth [5]. To cope with these stresses, plants have evolved various regulatory mechanisms at the molecular, cellular, physiological, and biochemical levels. This includes a myriad of transcription factors (TFs) to regulate stress-related gene expression, acting as a critical defense against abiotic stresses [6]. Based on existing research, 64 types of transcription factors have been discovered in plants [7], encompassing various families that participate in stress responses and regulate plant resistance. Examples include basic leucine zipper (bZIP) [8], basic helix-loop-helix (bHLH) [9], NAC [10], MYB [11], and WRKY [12].

bZIP transcription factors are among the most widely distributed and conserved gene families in higher plants and play a crucial role in many biotic and abiotic stresses [13,14]. The quantity of bZIP transcription factors differs among various plant species. In *Arabidopsis thaliana* [15], there are 78 identified members. In contrast, maize (*Zea mays* L.) [15], wheat (*Triticum aestivum* L.) [16], soybean (*Glycine max* L.) [17], and cotton (*Gossypium hirsutum* L.) [18] have a higher number of these factors, with 125, 187, 160, and 207 members, respectively. Research has shown that bZIP transcription factors participate in various biological processes, including regulating plant growth and development from seed germination [19], the growth and development of various organs like roots and stems [20,21,22,23], plant aging [23], light signal transduction [24], and responses to biotic [25,26,27] and abiotic stresses [28,29]. Under salt stress, bZIP transcription factors play a pivotal role in modulating the salt stress response through several pathways: First, they can either directly or indirectly activate responsive genes. For instance, in *Arabidopsis thaliana*, at least one TGACG motif exists within the upstream 1 kb of salt-responsive genes. The *AtbZIP17* protein in Arabidopsis has a high sequence homology with the TGA1b protein in tobacco (*Nicotiana tabacum*), which belongs to the TGA/OBF subfamily within the bZIP transcription factors. Both proteins can specifically bind to the TGACG motif, thereby regulating the expression of downstream genes [30]. Second, these transcription factors regulate genes that play a role in intracellular osmotic adjustment. The *OsbZIP71* protein in rice (*Oryza sativa*) specifically binds to the promoter of the *OsNHX1* gene (an antiporter protein in rice). The *OsNHX1* protein can transport excessive Na^+^ and K^+^ from the plant cytoplasm to the vacuoles. This enhances rice’s resistance to salt stress by adjusting cellular osmolarity [31]. Third, they participate in the removal of reactive oxygen species (ROS) within plants. The accumulation of ROS in plants is a clear indicator of oxidative stress when exposed to salt stress. In the *Chlamydomonas reinhardtii*, the bZIP transcription factor BLZ8 induces the carbon-concentrating mechanism (CCM) under oxidative stress. This increases the CO_2_ supply for photosynthesis, subsequently inhibiting ROS accumulation [32]. Moreover, when plants are under salt stress, apart from modulating intracellular K^+^/Na^+^ concentrations, the expression of plant bZIP transcription factors can also be influenced by plant hormones such as salicylic acid (SA), ethylene (ET), gibberellin (GA), methyl jasmonate/jasmonic acid (MeJA), and abscisic acid (ABA) [33].

Potassium (K^+^) is an essential macronutrient that plays significant roles in osmoregulation, membrane potential regulation, sugar cotransport, stress adaptation, and growth in plants [34,35]. K^+^ is very mobile within plant cells and can be reused repeatedly, meaning its efficiency relies heavily on a series of coordinated processes like absorption, transportation, storage, and redistribution [36]. Plants absorb K^+^ mainly through K^+^ channels and transport proteins located on the root cell plasma membranes [37]. The absorption of K^+^ is closely linked to root growth and structure, influencing cell expansion and root hair growth [38]. After absorption from the environment, K^+^ is transported and distributed to various tissues and organs via the plant’s vascular system [39]. K^+^ provides abiotic stress resistance. Under saline conditions, K^+^ maintains ionic balance and osmotic equilibrium, enhancing plant resilience to abiotic stresses [40,41]. Moreover, increasing K^+^ intake in plants activates the antioxidant defense system and reduces ROS accumulation, thus helping defend against abiotic stresses and significantly improving plant tolerance to such stresses [42]. Chen et al. reported that in *Tamarix* species, the uptake of K^+^ in roots under NaCl stress can elevate the activities of SOD, POD, and CAT, reducing the accumulation of ROS and enhancing the salt tolerance of the plant [43]. Similarly, studies by Song and Su demonstrated that in *Alternanthera philoxeroides*, the enrichment of K^+^ uptake under drought stress effectively alleviates the inhibitory effects of drought on the growth of the plant [44].

*Tamarix ramosissima* Ledeb (*T*. *ramosissima*) is one of the most widely distributed *Tamarix* species in the extreme arid regions of northwestern China, encompassing nearly all habitats occupied by *Tamarix* species [45]. *Tamarix* species are typical salt-secreting plants, possessing ‘salt glands’ made up of six secretory cells and two collecting cells [46]. These glands transport salts from within the plant to the surface, excreting them and thus protecting the plant from ion toxicity [47]. Studies by Lu et al. have shown that *T*. *ramosissima* thrives under salt concentrations less than 100 mM, but concentrations above 200 mM hinder its normal growth and development. This research aims to understand the response of bZIP transcription factors in *T*. *ramosissima* under a salt concentration of 200 mM and the application of KCl at the transcriptomic level. The goal is to identify key candidate genes regulated by bZIP transcription factors that mediate K^+^’s alleviation of salt stress and their critical metabolic pathways. This will provide a theoretical foundation for salt-resistant plant breeding and the role of bZIP transcription factors in mitigating salt damage.

## 2. Materials and Methods

### 2.1. Experimental Materials and Treatment

The experiment selected *T*. *ramosissima* cuttings with similar growth (5 months old) and placed them in a 24-hole hydroponic container filled with 1/2-strength Hoagland’s nutrient solution (size: 40 cm × 30 cm × 16 cm). The nutrient solution was replaced every 3 days. The cuttings were placed in a greenhouse with controlled conditions (temperature: 26 ± 2 °C, relative humidity: 40–55%) and grown for 2 months before use. The experiment was conducted in the laboratory of the College of Forestry at Nanjing Forestry University, with tamarisk seed sources coming from the Xinjiang Institute of Ecology and Geography, Chinese Academy of Sciences. Each experiment set up a control group and a treatment group, with 8 plants in each group and 3 replicates, totaling 24 plants. Plants were cultivated in 1/2-strength Hoagland’s nutrient solution as the control group. The treatment groups were cultivated in 1/2-strength Hoagland’s nutrient solution supplemented with 200 mM NaCl and another with 1/2-strength Hoagland’s nutrient solution supplemented with both 200 mM NaCl and 10 mM KCl. The nutrient solution was replaced every 3 days for all groups. The root samples were then collected at 0 h, 48 h, and 168 h after treatment for transcriptome sequencing.

### 2.2. Transcriptome Sequencing and Differential Gene Expression Screening

We selected root samples from treated *T*. *ramosissima* and sent them to a biotechnology company (GENE Denovo, Guangzhou, China) for transcriptome sequencing. RNA was isolated using the Invitrogen kit (Beinuo Bio, Shanghai, China). Following isolation, mRNA was enriched with oligo (dT) cellulose and then segmented to an average length of 200 nt with a fragmentation buffer from New England Biolabs (#E7530). Utilizing random hexamer primers, the first-strand cDNA synthesis occurred, succeeded by the creation of the second-strand cDNA using DNA polymerase I and RNase H. Subsequent steps included the purification of the cDNA fragments, the addition of a terminal “A” base, and their ligation to Illumina adapters. Size selection was conducted on an agarose gel, from which appropriate fragments were isolated and subjected to PCR amplification. The sequenced reads of these fragments were generated on the Illumina HiSeq system by a biotechnology company (GENE Denovo, Guangzhou, China) [48]. The raw data obtained from transcriptome sequencing was submitted to the Sequence Read Archive (SRA) database of the National Center for Biotechnology Information (NCBI) with an SRP number of SRP356215.

The read count data from sequencing were analyzed using DESeq2 software version 1.42.0 [49] to calculate the accurate False Discovery Rate (FDR) values, which are the *p*-values adjusted by the BH correction. Genes were considered significantly enriched if they had a corrected p-value of less than 0.05. Based on the differential analysis results, genes with an FDR < 0.05 and an absolute log_2_ fold-change (|log_2_FC|) greater than 1 (|log_2_FC| > 1) were identified as significant DEGs. We conducted DEG screening on the obtained data [50] and labeled them for the Gene Ontology (GO) [51] and Kyoto Encyclopedia of Genes and Genomes (KEGG) [52] for enrichment analysis.

### 2.3. Prediction of Pfam Protein Structure Domains

We aligned the protein sequences of the identified *bZIP* candidate genes in the Pfam database [53] to obtain labels for their protein domains. The basic physicochemical properties of the members of the bZIP gene family were analyzed and predicted online using Protparam (https://web.expasy.org/protparam/, accessed on 15 September 2023), including the molecular weight, theoretical pI, grand average of hydropathicity (GRAVY), instability index, aliphatic index, etc. Finally, the obtained bZIP proteins were subjected to subcellular localization prediction analysis using the website CELLOv.2.5 (http://cello.life.nctu.edu.tw/, accessed on 15 September 2023).

### 2.4. Validation of Candidate Genes with Quantitative Real-Time PCR (qRT-PCR)

We chose nine genes at random to validate the precision of our RNA-Seq results. We extracted the total RNA from *T. ramosissima* root samples using the RNAprep pure kit (Tiangeng, Beijing, China) and then synthesized cDNA with the Prime Script™ II 1st Strand cDNA Synthesis Kit (Takara, Beijing, China). The primers for this process were designed using the Primer Premier 5 software (Supplementary Table S1). For analysis, we utilized the SYBR Green Realtime PCR Master Mix (TOYBO, Jinan, China) and ran the samples on the ABI ViiA™ 7 Real-time PCR system (ABI, Carlsbad, CA, USA). The PCR amplification protocol included an initial denaturation at 95 °C for 30 s, followed by 40 cycles of denaturation at 95 °C for 5 s and annealing at 60 °C for 30 s. The melting curve process involved heating at 95 °C for 5 s, then 60 °C for 1 min, gradually increasing to 95 °C, and finally cooling to 50 °C for 30 s. Each gene underwent three biological replications, with Tubulin serving as the internal reference gene. We determined the relative expression levels using the 2^−ΔΔCt^ method [54].

### 2.5. Data Processing 

We processed the data using Microsoft Excel 2016 (Microsoft, Washington, DC, USA) to compute the mean, standard deviation, and log_2_ fold change. Phylogenetic trees were constructed using MEGA 11 software (MEGA software, State College, PA, USA). Statistical analyses, including ANOVA with the LSD post hoc test, were performed using SPSS 26.0 software (SPSS, New York, NY, USA). Graphical representations of the data were created with Origin 2019 software (OriginLab Corporation, Northampton, MA, USA).

## 3. Results

### 3.1. Transcriptome Data Quality Analysis

The obtained transcriptome data (Supplementary Table S2) show that RawDatas are within the range of 37,641,876–44,483,216. The percentage of CleanData is above 98%, Adapters are in the range of 0.25%–1.00%, and LowQuality is between 0.68% and 1.03%. The number of polyA reads (polyA) is 0, and the ratio of reads with N in high filtration (N) is between 0 and 0.01%. In summary, the quality of the transcriptome data in this study is relatively high and is suitable for subsequent analysis.

### 3.2. Identification and Physicochemical Property Analysis of bZIP Genes in the Roots of T. ramosissima

**Figure 1:**
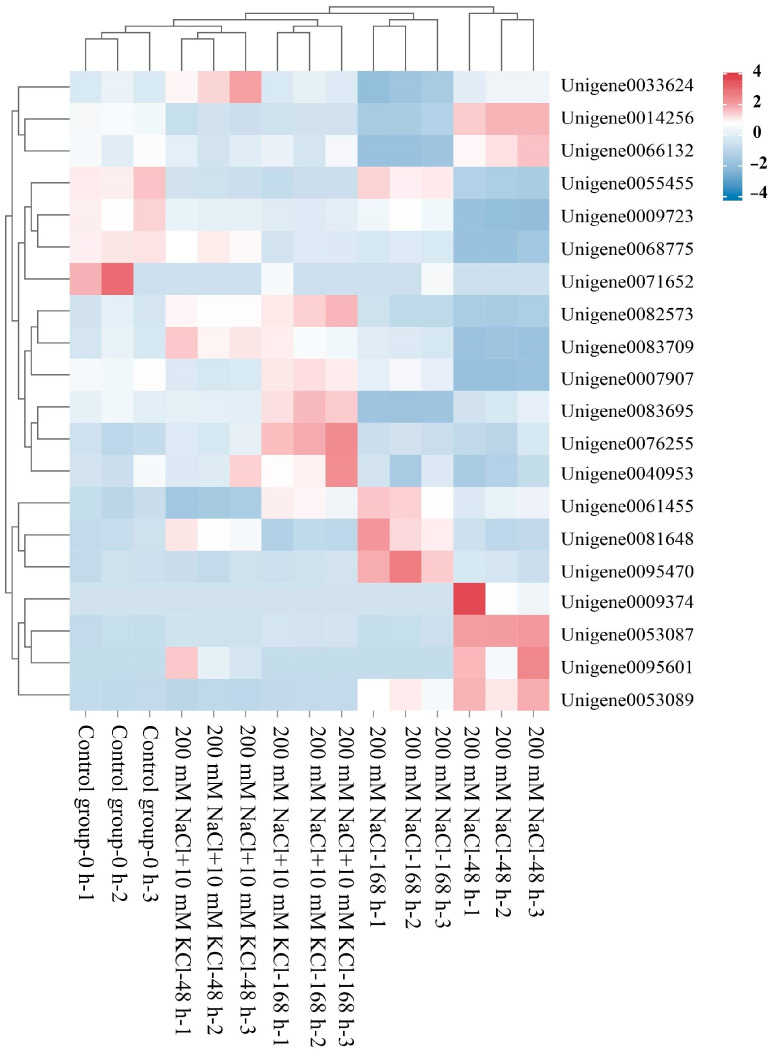
Clustering heatmap of the *bZIP* candidate genes (heatmap clustering analysis was performed on the 20 identified *bZIP* candidate genes across various samples).

Based on the gene label results from the transcriptome data in the NCBI database, we constructed a clustered heat map (Figure 1) for the *bZIP*-related genes and performed Pfam protein domain prediction and filtering (Supplementary Table S3). Ultimately, we identified bZIP transcription factors in the roots of the *T*. *ramosissima*. We used the Protparam online tool to analyze the physicochemical properties of the 20 identified bZIP transcription factors (Table 1). In this study, the clustered heat map clearly displays the distribution of these 20 *bZIP*-related genes across different samples. The results of the physicochemical property analysis show that the number of amino acids ranged from 75 (*bZIP*_13) to 749 (*bZIP*_9), showing a significant variation. The molecular weight ranged between 8828.02 (*bZIP*_13) and 80,805.18 (*bZIP*_9) Da, proportional to the number of amino acids. The theoretical pI ranged from 4.48 (*bZIP*_12) to 9.57 (*bZIP*_13). Among them, 10 members were basic proteins (pI > 7), and 10 members were acidic proteins (pI < 7). Only one bZIP (*bZIP*_20) had an instability index below 40, indicating that most of them are unstable proteins. The hydrophobicity results for all 20 bZIP proteins were less than 0, indicating that these bZIP proteins are hydrophilic. Subcellular localization predictions revealed that seven bZIP proteins (*bZIP*_1, *bZIP*_2, *bZIP*_11, *bZIP*_12, *bZIP*_13, *bZIP*_17, and *bZIP*_20) are located in the Cytoplasmic region, seven bZIP proteins (*bZIP*_4, *bZIP*_7, *bZIP*_8, *bZIP*_10, *bZIP*_14, *bZIP*_15, and *bZIP*_18) are located in the Extracellular region, three bZIP proteins (*bZIP*_6, *bZIP*_9, and *bZIP*_16) are in the Outer Membrane, and three bZIP proteins (*bZIP*_2, *bZIP*_5, and *bZIP*_19) are in the Periplasmic region.

### 3.3. Analysis of the Expression Levels of bZIP Genes in the Roots of T. ramosissima

Based on the expression level changes of these 20 bZIP genes (Supplementary Figure S1), three bZIP genes (bZIP_4, bZIP_8, and bZIP_11) showed an initial increase and then a decrease under 48 h and 168 h of NaCl stress. However, when additional KCl was added under NaCl stress, their expression levels first decreased and then increased. Two bZIP genes (bZIP_6 and bZIP_16) displayed a pattern of first decreasing and then increasing under 48 h and 168 h of NaCl stress, while their expression levels consistently rose when additional KCl was added under NaCl stress. Notably, the expression level of bZIP_17 consistently declined under 48 h and 168 h of NaCl stress but increased after an initial drop when additional KCl was added. In addition, bZIP_14 showed a constant increase in its expression level under both conditions, with a notably higher level when exogenous K^+^ was added compared to the control group.

### 3.4. Analysis of bZIP Genes Enriched in Gene Ontology and the Kyoto Encyclopedia of Genes and Genomes Pathway

Based on the GO enrichment of 20 *bZIP* genes obtained, the results (Supplementary Table S4) indicate that 5 *bZIP* genes are not enriched in GO, specifically *bZIP*_10, *bZIP*_13, *bZIP*_15, *bZIP*_19, and *bZIP*_20. Nine genes (*bZIP*_1, *bZIP*_2, *bZIP*_3, *bZIP*_6, *bZIP*_8, *bZIP*_11, *bZIP*_12, *bZIP*_14, and *bZIP*_17) are present in the GO Component. Fourteen genes (*bZIP*_1, *bZIP*_2, *bZIP*_3, *bZIP*_5, *bZIP*_6, *bZIP*_7, *bZIP*_8, *bZIP*_11, *bZIP*_12, *bZIP*_14, *bZIP*_16, *bZIP*_17, and *bZIP*_18) are found in the GO Function. Fifteen genes (*bZIP*_1, *bZIP*_2, *bZIP*_3, *bZIP*_4, *bZIP*_5, *bZIP*_6, *bZIP*_7, *bZIP*_8, *bZIP*_9, *bZIP*_11, *bZIP*_12, *bZIP*_14, *bZIP*_16, *bZIP*_17, and *bZIP*_18) are present in the GO Process.

Four *bZIP* genes (*bZIP*_2, *bZIP*_6, *bZIP*_16, and *bZIP*_18) are labeled in the plant hormone signal transduction pathway (Supplementary Table S4). In the N-48 h vs. N + K-48 h comparison group, *bZIP*_2 is downregulated, whereas *bZIP*_6, *bZIP*_16 and *bZIP*_18 are upregulated. In the same comparison group, only *bZIP*_6, *bZIP*_16, and *bZIP*_18 continue to be upregulated. Additionally, these four *bZIP* genes (*bZIP*_2, *bZIP*_6, *bZIP*_16, and *bZIP*_18) belong to the environmental information processing category in KEGG_A_class and to the signal transduction category in KEGG_B_class.

### 3.5. Analysis of the Plant Hormone Signal Transduction Pathway

Based on the differentially expressed genes labeled for the plant hormone signal transduction pathway when additional KCl was added under NaCl stress in the roots of *T*. *ramosissima* (Supplementary Table S5), 74 differentially expressed genes were observed in the 200 mM NaCl-48 h vs. 200 mM NaCl + 10 mM KCl-48 h (N-48 h vs. N + K-48 h) comparison group: 40 genes were upregulated, and 34 genes were downregulated. In the 200 mM NaCl-168 h vs. 200 mM NaCl + 10 mM KCl-168 h (N-168 h vs. N + K-168 h) comparison group, 70 differentially expressed genes were identified, with 40 genes upregulated and 30 genes downregulated. The plant hormone signal transduction pathway is classified under environmental information processing and was significantly enriched in differentially expressed genes in the N-48 h vs. N + K-48 h comparison group.

**Figure 2:**
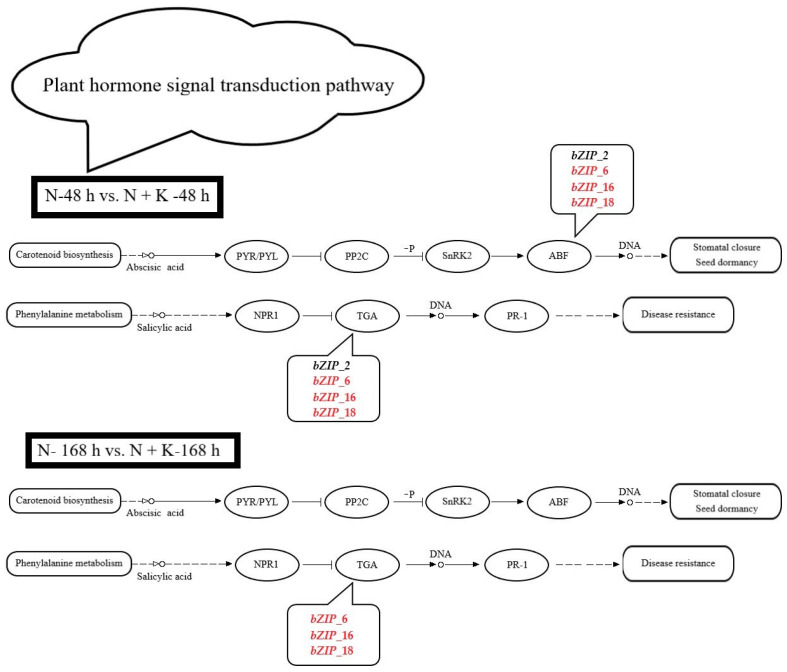
Label of *bZIP*-related differentially expressed genes in the plant hormone signal transduction pathway (changes in the *bZIP*-related DEGs labeled for the plant hormone signal transduction pathway in the roots of *T*. *ramosissima* under NaCl stress with the application of additional KCl for 48 h and 168 h. Red indicates differentially expressed genes that are upregulated, and black indicates differentially expressed genes that are downregulated).

According to Supplementary Figure S1 and Figure 2, four *bZIP* genes (*bZIP*_2, *bZIP*_6, *bZIP*_16, and *bZIP*_18) were labeled for the plant hormone signal transduction pathway, and their expression levels varied. Notably, in the N-48 h vs. N + K-48 h comparison group, *bZIP*_2 was downregulated, while *bZIP*_6, *bZIP*_16, and *bZIP*_18 were upregulated. In the N-168 h vs. N + K-168 h comparison group, the latter three genes continued to be upregulated. Based on the expression patterns of these four genes, we observed that under 200 mM NaCl for 48 h and 168 h, apart from *bZIP*_2, which first increased and then decreased, the other three genes’ expression levels first decreased and then increased. Interestingly, two genes (*bZIP*_6 and *bZIP*_16) consistently showed an upward trend under 48 h and 168 h of 200 mM NaCl + 10 mM KCl. However, *bZIP*_2 remained unchanged, while *bZIP*_18 first increased and then decreased.

### 3.6. Phylogenetic Analysis of Key Candidate bZIP Genes

Based on the *bZIP*-related genes obtained from the *T*. *ramosissima* under 48 h and 168 h with additional KCl applied during NaCl stress and their labels in the KEGG pathway regarding expression changes, we noticed that at a concentration of 200 mM NaCl for both 48 h and 168 h, the expression levels of two *bZIP*-related genes (*bZIP*_6 and *bZIP*_16) first decreased and then increased. Under 48 h and 168 h of 200 mM NaCl + 10 mM KCl, the expression levels of these two genes continually rose. Therefore, we speculate that these two genes (*bZIP*_6 and *bZIP*_16) are key candidate genes for the bZIP transcription factor.

**Figure 3:**
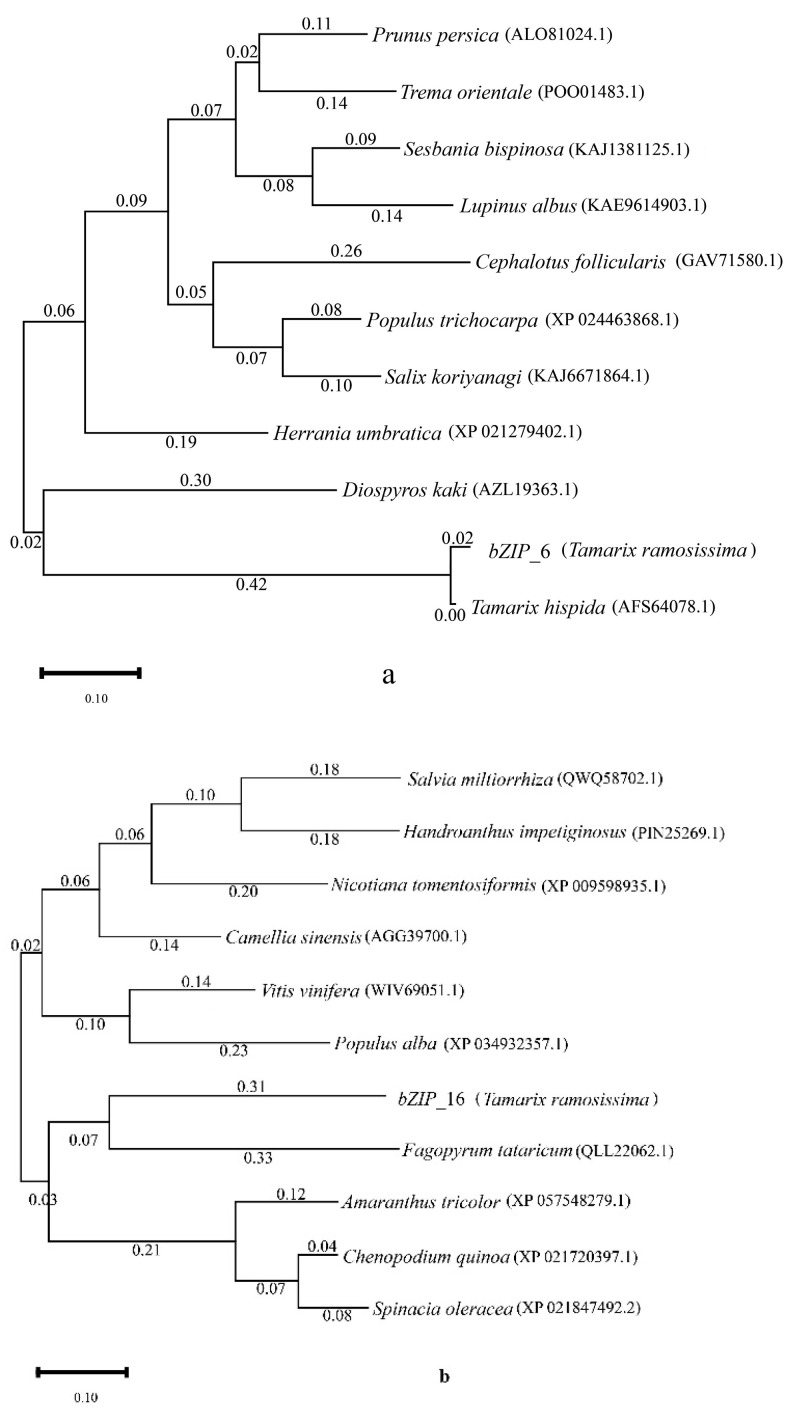
Phylogenetic tree analysis of *T*. *ramosissima* bZIP genes and bZIP genes of other species. (Analysis of the phylogenetic tree constructed from the protein amino acid sequences of *bZIP*_6 and *bZIP*_16 from *T*. *ramosissima* roots, along with those of 10 other homologous gene species. (**a**) Evolutionary tree constructed for the *bZIP*_6 protein. (**b**) Evolutionary tree constructed for the *bZIP*_16 protein.)

We selected these two key candidate genes (*bZIP*_6 and *bZIP*_16) and used their protein amino acid sequences to perform a BLAST search on the NCBI website. We then chose ten homologous species for each gene (Supplementary Table S6) and constructed a phylogenetic tree. The results showed that *bZIP*_6 is closely related to *Tamarix hispida* (*T*. *hispida*), while *bZIP*_16 has a close phylogenetic relationship with *Fagopyrum tataricum* (Figure 3).

### 3.7. qRT-PCR Validation of bZIP Candidate Genes

**Figure 4:**
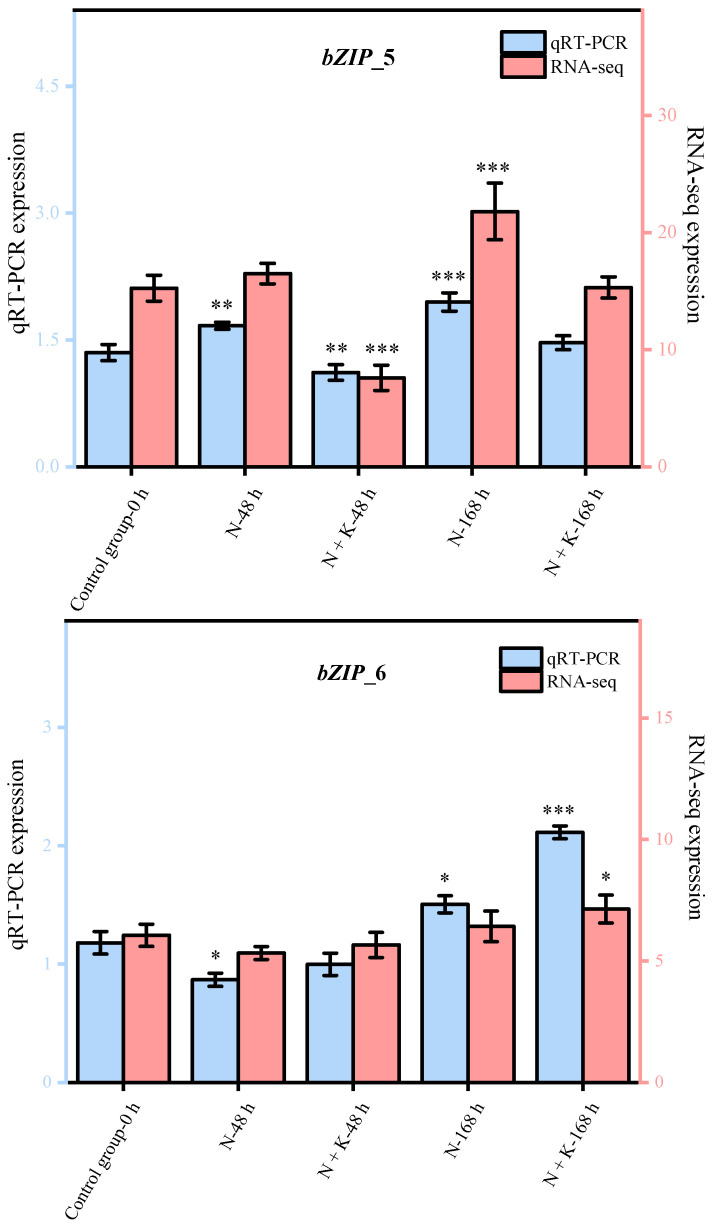

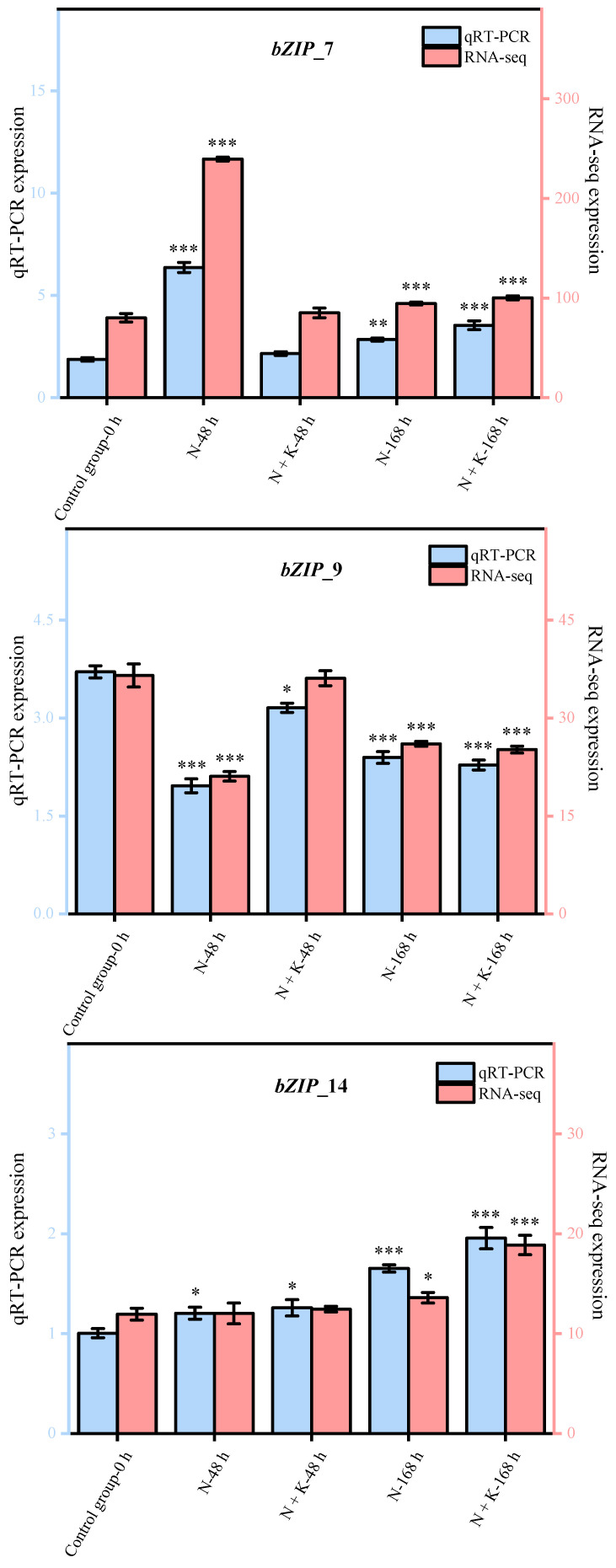

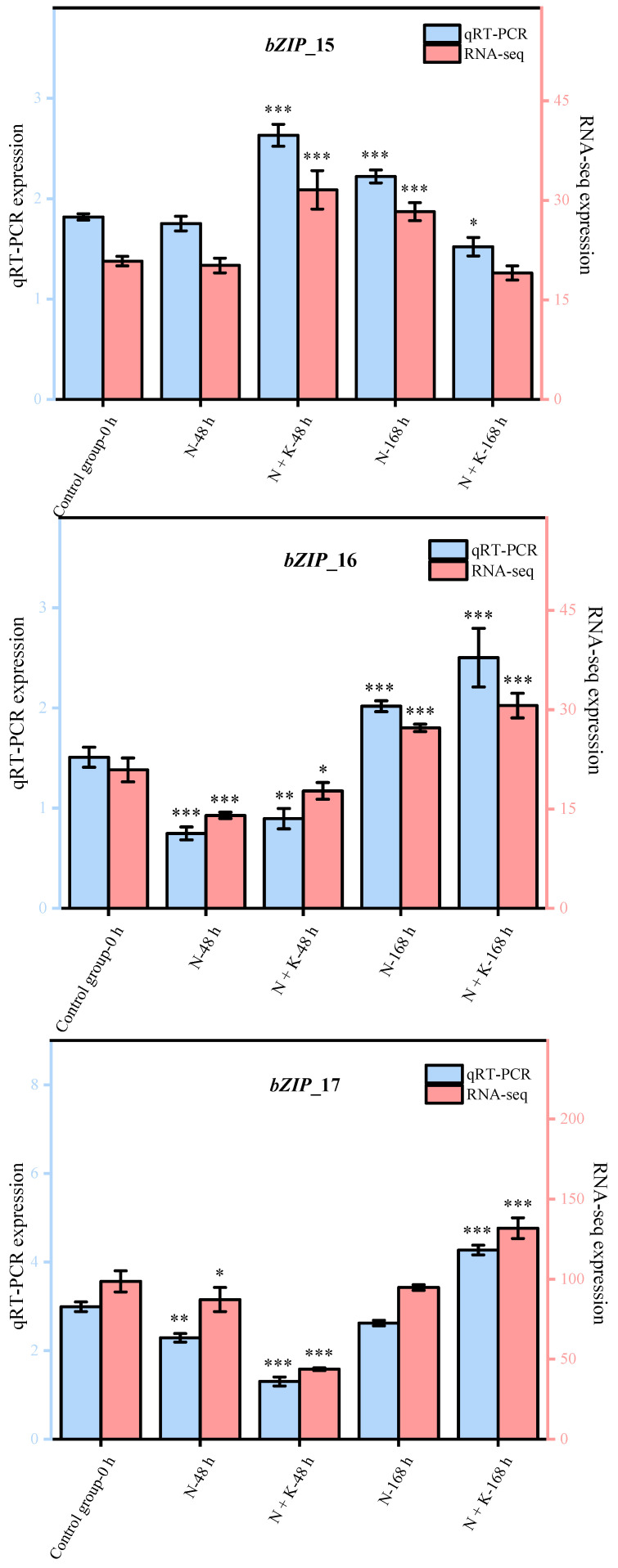

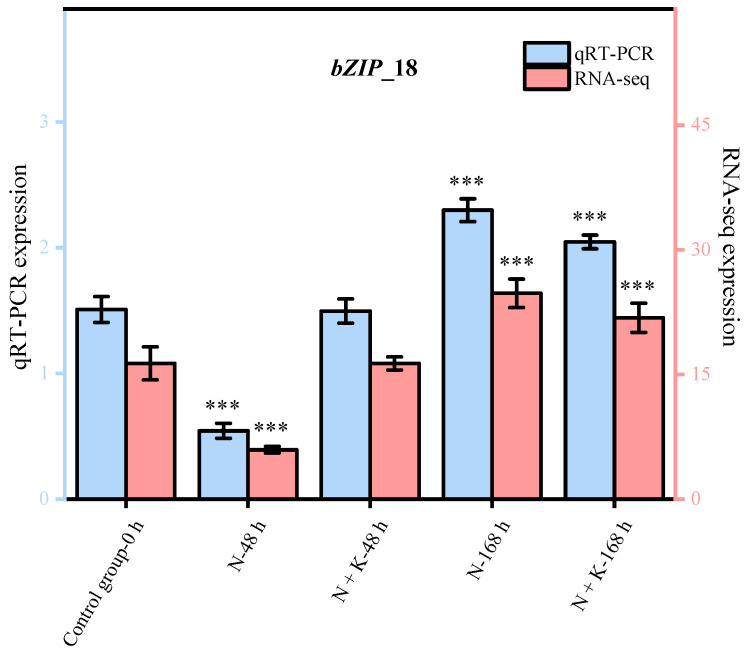
Quantitative real-time PCR validation of *bZIP* candidate key genes. (Nine DEGs were randomly selected for qRT-PCR validation, and the error bars were obtained from multiple replicates of qRT-PCR. Note: *p* ≥ 0.05 is not marked; 0.01 < *p* < 0.05 is marked as *; 0.001 < *p* < 0.01 is marked as **; *p* ≤ 0.001 is marked as ***; 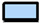: Numerical value has been shown on the left side of the *Y* axis; 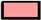: Numerical value has been shown on the right side of the *Y* axis).

Nine principal bZIP genes, chosen at random from a group of 20, underwent qRT-PCR analysis. The data from this qRT-PCR were employed to corroborate the precision of the transcriptome sequencing findings in our research. It was observed that the expression patterns of these nine genes, as determined by qRT-PCR, aligned with the trends noted in the transcriptome sequencing analysis (refer to Figure 4). This alignment offers ample proof of the dependability and exactness of the transcriptome sequencing outcomes in this investigation.

## 4. Discussion

bZIP transcription factors constitute one of the largest and most diverse families in the plant kingdom, with a wide distribution across eukaryotes [55,56]. They are integral to numerous processes in plant biology, including growth, seed development, secondary metabolism, and responses to various stresses [57,58]. Notably, they play a pivotal regulatory role in enhancing plant resistance to abiotic stresses [59,60]. Across different species, bZIP gene family members have been identified in varying numbers, ranging from 62 in the watermelon [61] to 160 in the soybean [17], with counts such as 64 in the yellowhorn [62], 55 in the grapevine [63], and 112 in the apple [64].

However, due to the incomplete genome sequencing of *T*. *ramosissima*, there has been limited research on the identification of bZIP gene family members within this plant species. In this study, we conducted transcriptome sequencing to identify 20 members of the bZIP gene family in *T*. *ramosissima* in response to NaCl stress with additional KCl applied. Our comprehensive analysis of these 20 *bZIP* genes contributes to a deeper understanding of the salt-tolerant genes associated with bZIP in *T*. *ramosissima* under the influence of additional KCl supplementation during NaCl stress. This knowledge provides a robust scientific basis for the breeding of salt-tolerant crop varieties tailored for saline-alkali areas. The non-biological stress response network mediated by bZIP transcription factors is a crucial mechanism for plants to respond to various abiotic stresses [65]. Under NaCl conditions, *ThbZIP1* in *T*. *hispida* might regulate the ROS scavenging capacity and stress tolerance-related physiological changes by activating stress-tolerant genes, thus achieving a non-biological stress tolerance and playing a crucial role in ABA-mediated *T*. *hispida* stress responses [66]. Additionally, under salt stress, overexpression of *ThbZIP1* can enhance the activity of peroxidase (POD) and superoxide dismutase (SOD), and *ThbZIP1* enhances ROS scavenging, promotes the accumulation of compatible solutes, induces and strengthens the biosynthesis of soluble proteins, granting plants resistance [67]. In this study, under 200 mM NaCl conditions for 48 h and 168 h, the expression levels of two *bZIP*-related genes (*bZIP*_6 and *bZIP*_16) first decreased and then increased, and the expression level of one bZIP-related gene (*bZIP*_14) has been rising. Notably, the activities of SOD, POD, and CAT in *T*. *ramosissima* under 200 mM NaCl conditions with exogenous K^+^ for 48 h and 168 h increased, and the antioxidant enzyme system was activated to remove the ROS produced by high salt stress to resist NaCl stress, enhancing plant salt tolerance [43]. Under the influence of additional KCl under the NaCl conditions in this study, the expression levels of *bZIP*_6, *bZIP*_16, *bZIP*_7, and *bZIP*_14 under exogenous K^+^ for 48 h and 168 h under NaCl stress were always found to be on the rise. The results suggest that these *bZIP*-related genes in *T*. *ramosissima* may improve plant antioxidant enzyme activity by upregulating expression levels under exogenous K^+^ in response to NaCl stress and clear ROS accumulation, building an antioxidant defense system and enhancing plant salt tolerance.

ABF transcription factors (ABA-responsive element binding factors) are a type of bZIP protein that specifically recognizes ABRE and belongs to subgroup A of the bZIP family. They mainly participate in the regulation of ABA and stress responses and are also known as ABA-responsive element binding proteins (AREBs) or ABRE binding factors (ABFs) [68,69,70]. ABRE is an ABA response element, and bZIP transcription factors can bind to ABRE elements, known as ABA response element-binding factors, which play a significant role in the ABA-dependent signaling pathway [71]. bZIP is a typical ABRE binding transcription factor, which binds to the ABRE element and trans-activates downstream gene expression [72]. In Tartary buckwheat (*Fagopyrum tataricum*), the *FtbZIP5* gene homologous to ABF was induced by salt stress, and its overexpression in Arabidopsis reduced oxidative damage to transgenic plants under salt stress. Moreover, *FtbZIP5*, as a positive regulator, mediated plant tolerance to salt and drought through ABA-dependent signaling pathways [73]. In this study, four *bZIP*-related genes were labeled for the plant hormone signal transduction pathway and activated gene expression. These *bZIP* genes, induced by ABA, participated in the regulation of NaCl stress. Among them, in the comparison group of N-48 h vs. N + K-48 h, *bZIP*_2 was downregulated, while *bZIP*_6, *bZIP*_16, and *bZIP*_18 were upregulated. According to the GO classification system, the *bZIP* genes *bZIP*_2, *bZIP*_6, *bZIP*_16, and *bZIP*_18 are enriched in GO categories, indicating their involvement in biological processes, molecular functions, and cellular components. From this, it can be inferred that these *bZIP*-related genes in the roots of *T*. *ramosissima* mediate the plant’s tolerance to NaCl through the ABA signaling pathway, thereby enhancing the plant’s inherent salt resistance. 

Furthermore, Salicylic acid (SA) is an important plant hormone that participates in the plant’s response to stress through complex signal transduction networks [74]. Plant bZIP-type transcription factors are induced in their expression by SA [75]. In the plant hormone signal transduction pathway, SA primarily mediates the signaling pathway to establish an efficient defense response mechanism against the invasion of pathogens [76]. SA-mediated disease resistance pathways in plants are closely associated with TGA [77]. In this study, three *bZIP*-related genes (*bZIP*_6, *bZIP*_16, and *bZIP*_18) consistently showed upregulated expression levels in response to SA induction after 48 h and 168 h of exposure to exogenous potassium under NaCl stress.

In summary, as mentioned above, both *bZIP*_6 and *bZIP*_16, as *bZIP*-related genes, consistently showed a trend of upregulation in their expression levels after 48 h and 168 h of exogenous potassium addition under NaCl stress. Furthermore, they are induced by both ABA and SA in the plant hormone signal transduction pathway. Their upregulated expression plays a crucial role when their signal pathways are activated. From this, it is suggested that *bZIP*_6 and *bZIP*_16 can be regarded as key candidate genes related to bZIP, and their functions can be further verified. In conclusion, the random selection of nine key candidate *bZIP* genes for validation has confirmed the reliability and accuracy of the transcriptome data in this study.

## 5. Conclusions

The bZIP protein family plays a crucial role in plant growth and resisting adverse environmental stresses. In this study, under NaCl stress, after exogenous K^+^ was applied to the roots of *T*. *ramosissima* for 48 h and 168 h, we found that bZIP transcription factors were involved in constructing defense systems through related metabolic pathways to combat environmental adversity. They likely mediate signal transduction through various physiological pathways, clear the accumulation of ROS, reduce plant oxidative damage, and thus resist NaCl stress. Notably, adding K^+^ greatly enhances the plant’s K^+^/Na^+^ ratio, helping it combat the stress caused by NaCl. Additionally, K^+^ plays a vital role in maintaining the normal growth of *T*. *ramosissima*. Furthermore, bZIP transcription factors, mediated by the ABA signaling pathway in the plant hormone signal transduction pathway, enhance the salt resistance of *T*. *ramosissima*. Especially, *bZIP*_6 and *bZIP*_16, as *bZIP*-related genes, were labeled by the plant hormone signal transduction pathway under NaCl stress with the addition of exogenous K^+^ at 48 h and 168 h, and their expression consistently showed an upward trend. Hence, they might play a vital role as key candidate genes associated with bZIP in resisting NaCl damage. However, the regulatory mechanisms, metabolic products, and functional verification for alleviating NaCl toxicity by these genes still require further investigation.

## Figures and Tables

**Table 1:** Physicochemical properties of bZIP protein in *Tamarix ramosissima*.

ID	Gene	ORF/aa	Molecular Weight	Theoretical pI	GRAVY	Instability Index	Aliphatic Index	Subcellular Localization
*bZIP*_1	*Unigene0007907*	144	16,481.66	5.85	−0.59	59.09	84.58	Cytoplasmic
*bZIP*_2	*Unigene0009374*	198	22,629.33	6.19	−0.78	76.79	74.44	Periplasmic
*bZIP*_3	*Unigene0009723*	144	16,486.73	6.60	−0.61	57.27	83.26	Cytoplasmic
*bZIP*_4	*Unigene0014256*	170	18,755.94	8.10	−0.65	65.76	74.06	Extracellular
*bZIP*_5	*Unigene0033624*	230	26,706.5	7.23	−1.13	73.83	57.70	Periplasmic
*bZIP*_6	*Unigene0040953*	243	26,881.25	5.62	−0.75	61.91	67.00	OuterMembrane
*bZIP*_7	*Unigene0053087*	181	19,900.05	5.93	−0.78	57.88	52.82	Extracellular
*bZIP*_8	*Unigene0053089*	172	18,846.27	7.89	−0.53	57.71	72.03	Extracellular
*bZIP*_9	*Unigene0055455*	749	80,805.18	7.21	−0.62	42.51	66.52	OuterMembrane
*bZIP*_10	*Unigene0061455*	581	63,750.72	7.71	−0.81	67.55	62.48	Extracellular
*bZIP*_11	*Unigene0066132*	224	25,914.92	6.13	−0.60	73.63	80.09	Cytoplasmic
*bZIP*_12	*Unigene0068775*	344	38,475.14	4.48	−0.44	62.21	88.2	Cytoplasmic
*bZIP*_13	*Unigene0071652*	75	8828.02	9.57	−1.113	49.75	80.67	Cytoplasmic
*bZIP*_14	*Unigene0076255*	278	29,558.58	7.38	−0.89	59.65	54.21	Extracellular
*bZIP*_15	*Unigene0081648*	509	55,507.17	8.75	−0.91	67.7	52.73	Extracellular
*bZIP*_16	*Unigene0082573*	491	53,591.74	7.85	−0.64	53.35	66.58	OuterMembrane
*bZIP*_17	*Unigene0083695*	134	15,425.31	5.46	−0.77	76.81	77.91	Cytoplasmic
*bZIP*_18	*Unigene0083709*	457	49,264.29	9.23	−0.87	46.82	57.26	Extracellular
*bZIP*_19	*Unigene0095470*	339	37,936.96	5.27	−0.90	66.77	59.97	Periplasmic
*bZIP*_20	*Unigene0095601*	194	21,430.75	6.53	−0.26	30.37	97.27	Cytoplasmic

Note: ORF means open reading frame; GRAVY means the grand average of hydropathicity.

## Data Availability

Data are contained within the article and Supplementary Materials.

## Supplementary Materials

The following supporting information can be downloaded at: https://www.mdpi.com/article/10.3390/genes14122203/s1, Supplementary Table S1: Sequences of specific primers; Supplementary Table S2: Data filtering statistics table; Supplementary Table S3: Pfam prediction of protein domain structures in *bZIP* candidate genes; Supplementary Table S4: Analysis of *bZIP* genes enriched in GO and KEGG pathway; Supplementary Table S5: Analysis of plant hormone signal transduction pathway; Supplementary Table S6. Information sheet for the 10 homologous gene species; Supplementary Figure S1: Changes in the expression level of *bZIP* genes.
