# Supplementary material for: Role of bZIP Transcription Factors in Response to NaCl Stress in Tamarix ramosissima under Exogenous Potassium (K+)
Source: Genes (Basel). 2023 Dec 13;14(12):2203. doi: 10.3390/genes14122203 (PMC10743189; doi:10.3390/genes14122203)
Supplement: Supplementary file 1 [file genes-14-02203-s001.zip › genes-2706441-supplementary.pdf]

Supplementary Table S1. Sequences of specific primers

ID	Primer name	Primer sequence (5'-3')
*bZIP*_16	*Unigene0082573*	F: CGGGGTTGTCATGGAGCATA<br>R: CAGCACCTTGATTGGGTTGC
*bZIP*_6	*Unigene0040953*	F: TGGCAAAGACAGAGGTCGAA<br>R: AAGGTGCAGGTGATAAGCCC
*bZIP*_7	*Unigene0053087*	F: ACAAGCATGAATGTCACGGC<br>R: CGCAAAAGTTCTCCTCGTCC
*bZIP*_17	*Unigene0083695*	F: GCTTCCGCACTTGGGATTTG<br>R: TCTTGACCGTCGAGCTGATTC
*bZIP*_14	*Unigene0076255*	F: ATGGCCCCCAGAATGGTTTC<br>R: TGCATAGCAGGTATCGCAGG
*bZIP*_18	*Unigene0083709*	F: AGTCAGCATCAGGAACCACG<br>R: TTCCTGAACAACCCCTGCTC
*bZIP*_5	*Unigene0033624*	F: TCATGCCGGTTCGGATTTGA<br>R: AGAGCTCGAGTTCGGAGAGT
*bZIP*_15	*Unigene0081648*	F: AATGCACAGGCGGTCGATAA<br>R: AGGCTTACCGGACTCAGGAT
*bZIP*_9	*Unigene0055455*	F: GCGCCAAATCGTCCGTTATC<br>R: CTATGACCCGAATCGGCCTG
	Tubulin	F: GCTGAGATTACAACCGCTG<br>R: CTGTTCGTTTGGTCTTGATT

Note: F means Forward primer, R means Reversed primer.

Supplementary Table S2. Data filtering statistics table

Sample	RawDatas	CleanData(%)	Adapter(%)	LowQuality(%)	polyA(%)	N(%)
Control group-0 h-1	43090746	42525274 (98.69%)	252204 (0.59%)	308850 (0.72%)	0 (0.00%)	4418 (0.01%)
Control group-0 h-2	43083210	42264990 (98.10%)	429632 (1.00%)	383916 (0.89%)	0 (0.00%)	4672 (0.01%)
Control group-0 h-3	41837770	41301300 (98.72%)	233664 (0.56%)	298868 (0.71%)	0 (0.00%)	3938 (0.01%)
N-48 h-1	37641876	37103264 (98.57%)	152462 (0.41%)	386150 (1.03%)	0 (0.00%)	0 (0.00%)
N-48 h-3	43708134	43242360 (98.93%)	133820 (0.31%)	331950 (0.76%)	0 (0.00%)	4 (0.00%)
N-48 h-3	44840066	44378790 (98.97%)	156148 (0.35%)	305128 (0.68%)	0 (0.00%)	0 (0.00%)
N-168 h-1	43816684	43212896 (98.62%)	157908 (0.36%)	445870 (1.02%)	0 (0.00%)	10 (0.00%)
N-168 h-2	44021530	43498298 (98.81%)	107958 (0.25%)	415272 (0.94%)	0 (0.00%)	2 (0.00%)
N-168 h-3	44483216	43938688 (98.78%)	129992 (0.29%)	414536 (0.93%)	0 (0.00%)	0 (0.00%)
N + K-48 h-1	42157406	41555604 (98.57%)	281692 (0.67%)	316144 (0.75%)	0 (0.00%)	3966 (0.01%)
N + K-48 h-2	42321718	41683754 (98.49%)	284526 (0.67%)	349468 (0.83%)	0 (0.00%)	3970 (0.01%)
N + K-48 h-3	42750232	42055378 (98.37%)	359420 (0.84%)	331168 (0.77%)	0 (0.00%)	4266 (0.01%)
N + K-168 h-1	42104080	41530626 (98.64%)	252242 (0.60%)	317294 (0.75%)	0 (0.00%)	3918 (0.01%)
N + K-168 h-2	42091546	41435868 (98.44%)	276730 (0.66%)	375140 (0.89%)	0 (0.00%)	3808 (0.01%)
N + K-168 h-3	43098086	42449748 (98.50%)	320946 (0.74%)	323192 (0.75%)	0 (0.00%)	4200 (0.01%)

Note: N-48 h: 200 mM NaCl-48 h, N + K-48 h: 200 mM NaCl + 10 mM KCl-48 h, N-168 h: 200 mM NaCl-168 h, N + K-168 h: 200 mM NaCl + 10 mM KCl-168 h.

Supplementary Table S3. Pfam prediction of protein domain structures in *bZIP* candidate genes

ID	Gene	envelope start	envelope end	hmm acc	hmm length	bit score	clan	PfamA_definition
*bZIP*_1	*Unigene0007907*	18	80	PF00170.21	64	47.1	CL0018	bZIP transcription factor
*bZIP*_2	*Unigene0009374*	81	128	PF00170.21	64	31.2	CL0018	bZIP transcription factor
*bZIP*_3	*Unigene0009723*	19	80	PF00170.21	64	44	CL0018	bZIP transcription factor
*bZIP*_4	*Unigene0014256*	17	70	PF00170.21	64	37.4	CL0018	bZIP transcription factor
*bZIP*_5	*Unigene0033624*	124	179	PF00170.21	64	38.6	CL0018	bZIP transcription factor
*bZIP*_6	*Unigene0040953*	189	239	PF00170.21	64	47.8	CL0018	bZIP transcription factor
*bZIP*_7	*Unigene0053087*	32	93	PF00170.21	64	43.3	CL0018	bZIP transcription factor
*bZIP*_8	*Unigene0053089*	34	95	PF00170.21	64	39.4	CL0018	bZIP transcription factor
*bZIP*_9	*Unigene0055455*	263	326	PF00170.21	64	43.6	CL0018	bZIP transcription factor
*bZIP*_10	*Unigene0061455*	434	495	PF00170.21	64	34.5	CL0018	bZIP transcription factor
*bZIP*_11	*Unigene0066132*	80	141	PF00170.21	64	49.7	CL0018	bZIP transcription factor
*bZIP*_12	*Unigene0068775*	175	238	PF00170.21	64	36.5	CL0018	bZIP transcription factor
*bZIP*_13	*Unigene0071652*	40	75	PF00170.21	64	39.2	CL0018	bZIP transcription factor
*bZIP*_14	*Unigene0076255*	209	272	PF00170.21	64	72.6	CL0018	bZIP transcription factor
*bZIP*_15	*Unigene0081648*	428	490	PF00170.21	64	38	CL0018	bZIP transcription factor
*bZIP*_16	*Unigene0082573*	400	447	PF00170.21	64	47.3	CL0018	bZIP transcription factor
*bZIP*_17	*Unigene0083695*	25	81	PF00170.21	64	37.4	CL0018	bZIP transcription factor
*bZIP*_18	*Unigene0083709*	411	457	PF00170.21	64	44.2	CL0018	bZIP transcription factor
*bZIP*_19	*Unigene0095470*	260	330	PF00170.21	64	32.3	CL0018	bZIP transcription factor
*bZIP*_20	*Unigene0095601*	1	38	PF00170.21	64	29.9	CL0018	bZIP transcription factor

Note: envelope start: HMM model predicted structural domain start position of Unigene coding protein sequence, envelope end: HMM model predicted structural domain end position of Unigene coding protein sequence, hmm acc: Unigene encodes the number of the HMM corresponding to the structure of the protein sequence in Pfam, hmm length: length of matching sequence in the database, bit score: score of Unigene coding protein sequence structure compared to HMM model, clan: classification of Unigene coding protein sequence by protein sequence, structure and HMM file in the Pfam database, PfamA_definition: the name of the structure corresponding to the query sequence in PfamA.

Supplementary Table S4. Analysis of bZIP genes enriched in GO and KEGG pathway

ID	Description	KEGG_A_class	KEGG_B_class	Pathway	GO Component	GO Function	GO Process	Log_2 fold change	
								N-48 h vs. N + K-48 h	N-168 h vs. N + K-168 h
*bZIP*_1	bZIP transcription factor	-	-	-	intracellular membrane-bounded organelle	nucleic acid binding transcription factor activity; DNA binding; protein dimerization activity	regulation of transcription, DNA-templated; protein targeting; seed maturation; cellular response to stress	0.94	0.29
*bZIP*_2	bZIP transcription factor	Environmental Information Processing	Signal transduction	ko04075	intracellular membrane-bounded organelle	nucleic acid binding transcription factor activity; DNA binding	signal transduction; gene expression; cellular macromolecule biosynthetic process; response to chemical; floral organ formation; regulation of post-embryonic development	-9.55	0.00
*bZIP*_3	bZIP transcription factor	-	-	-	intracellular membrane-bounded organelle	nucleic acid binding transcription factor activity; DNA binding; protein dimerization activity	regulation of transcription, DNA-templated; protein targeting; seed maturation; cellular response to stress	1.22	-0.22
*bZIP*_4	bZIP transcription factor 11	-	-	-	-	-	regulation of gene expression; regulation of cellular process	-0.71	0.43
*bZIP*_5	bZIP5	-	-	-	-	DNA binding	signal transduction; response to chemical	0.40	1.01
*bZIP*_6	bZIP transcription factor ABF8	Environmental Information Processing	Signal transduction	ko04075	intracellular membrane-bounded organelle	DNA binding	response to acid chemical; regulation of transcription, DNA-templated; signal transduction; response to hormone; positive regulation of biological process; cellular response to organic substance	0.27	0.34

*bZIP*_7	Predicted: bZIP transcription factor 11	-	-	-	-	DNA binding; protein dimerization activity	regulation of gene expression; regulation of cellular process	-1.34	0.24
*bZIP*_8	Predicted: bZIP transcription factor 11	-	-	-	intracellular membrane-bounded organelle	nucleic acid binding transcription factor activity; protein dimerization activity	signal transduction; negative regulation of translation in response to stress; response to disaccharide	-2.60	-1.79
*bZIP*_9	bZIP transcription factor 17-like	-	-	-	photosystem; plastid stroma; plastid thylakoid lumen	-	regulation of transcription, DNA-templated; hyperosmotic response; plastid membrane organization; organ morphogenesis; response to chemical; cellular developmental process	0.31	-0.52
*bZIP*_10	bZIP8	-	-	-	-	-	-	-0.61	-0.12
*bZIP*_11	bZIP11	-	-	-	intracellular membrane-bounded organelle	nucleic acid binding transcription factor activity; DNA binding; protein dimerization activity	regulation of gene expression	-0.81	5.22
*bZIP*_12	bZIP12	-	-	-	intracellular membrane-bounded organelle; membrane part	DNA binding	immune system process; defense response; response to temperature stimulus; hormone-mediated signaling pathway; gene expression; cellular response to stress; cellular macromolecule biosynthetic process; intracellular signal transduction; regulation of programmed cell death; response to other organism; cellular response to acid chemical; cellular response to oxygen-containing compound	1.27	-0.01
*bZIP*_13	bZIP transcription factor 17-like isoform X1	-	-	-	-	-	-	0.00	0.06

*bZIP*_14	bZIP transcription factor 16 isoform X3	-	-	-	intracellular membrane-bounded organelle	nucleic acid binding transcription factor activity; DNA binding	regulation of gene expression; cellular macromolecule biosynthetic process	0.18	0.60
*bZIP*_15	bZIP8	-	-	-	-	-	-	0.49	-0.73
*bZIP*_16	bZIP transcription factor TRAB1-like	Environmental Information Processing	Signal transduction	ko04075	-	DNA binding	response to acid chemical; regulation of transcription, DNA-templated; response to osmotic stress; hormone-mediated signaling pathway	0.96	0.79
*bZIP*_17	bZIP transcription factor 44-like	-	-	-	intracellular membrane-bounded organelle	nucleic acid binding transcription factor activity; DNA binding; protein dimerization activity	signal transduction; negative regulation of translation in response to stress; response to disaccharide	0.12	1.59
*bZIP*_18	bZIP14	Environmental Information Processing	Signal transduction	ko04075	-	nucleic acid binding	response to acid chemical; developmental process involved in reproduction; transcription, DNA-templated; response to stress; signal transduction; response to abiotic stimulus; response to hormone; post-embryonic development; system development; cellular response to organic substance; cellular response to oxygen-containing compound	2.06	0.42
*bZIP*_19	BZIP-type transcription factor MBZ1-like	-	-	-	-	-	-	-1.52	-3.29
*bZIP*_20	BZIP domain-containing protein, partial	-	-	-	-	-	-	-0.96	0.00

Note：N-48 h: 200 mM NaCl-48 h, N + K-48 h: 200 mM NaCl + 10 mM KCl-48 h, N-168 h: 200 mM NaCl-168 h, N + K-168 h: 200 mM NaCl + 10 mM KCl-168 h; ko04075: Plant hormone signal transduction.

Supplementary Table S5. Analysis of Plant hormone signal transduction pathway

Pathway	Gene numbers	Class	*p*-Value	Up	Down
N-48 h vs. N + K-48 h					
Plant hormone signal transduction pathway	74	Environmental Information Processing	0.000079	40	34
N-168 h vs. N + K-168 h					
Plant hormone signal transduction pathway	70	Environmental Information Processing	0.186311	40	30

Note: N-48 h: 200 mM NaCl-48 h, N + K-48 h: 200 mM NaCl + 10 mM KCl-48 h, N-168 h: 200 mM NaCl-168 h, N + K-168 h: 200 mM NaCl + 10 mM KCl-168 h.

Supplementary Table S6. Information sheet for the 10 homologous gene species

Number	Family	Species	Description	Protein ID	CDS（bp）	ORF length（aa）
bZIP_6						
1	Tamaricaceae	*Tamarix hispida*	bZIP transcription factor ABF8	AFS64078.1	756	251
2	Ebenaceae	*Diospyros kaki*	transcription factor bZIP5	AZL19363.1	753	250
3	Salicaceae	*Populus trichocarpa*	bZIP transcription factor TRAB1 isoform X1	XP_024463868.1	831	276
4	Malvaceae	*Herrania umbratica*	bZIP transcription factor 23-like isoform X2	XP_021279402.1	816	271
5	Rosaceae	*Prunus persica*	bZIP protein 5	ALO81024.1	813	270
6	Salicaceae	*Salix koriyanagi*	bZIP transcription factor, partial	KAJ6671864.1	717	238
7	Cannabaceae	*Trema orientale*	bZIP transcription factor	POO01483.1	798	265
8	Fabaceae	*Sesbania bispinosa*	bZIP domain	KAJ1381125.1	735	244
9	Cephalotaceae	*Cephalotus follicularis*	bZIP_1 domain-containing protein	GAV71580.1	1080	359
10	Fabaceae	*Lupinus albus*	putative transcription factor bZIP family	KAE9614903.1	810	269
bZIP_16						
1	Theaceae	*Camellia sinensis*	bZIP transcription factor bZIP8	AGG39700.1	1311	436
2	Amaranthaceae	*Chenopodium quinoa*	bZIP transcription factor 23-like	XP_021720397.1	1479	492
3	Vitaceae	*Vitis vinifera*	bZIP transcription factor 45	WIV69051.1	1344	447
4	Polygonaceae	*Fagopyrum tataricum*	bZIP83	QLL22062.1	1344	447
5	Amaranthaceae	*Amaranthus tricolor*	bZIP transcription factor TRAB1-like	XP_057548279.1	1401	466
6	Amaranthaceae	*Spinacia oleracea*	bZIP transcription factor TRAB1	XP_021847492.2	1452	483
7	Lamiaceae	*Salvia miltiorrhiza*	bZIP2	QWQ58702.1	1386	461
8	Solanaceae	*Nicotiana tomentosiformis*	bZIP transcription factor 46-like	XP_009598935.1	1329	442
9	Bignoniaceae	*Handroanthus impetiginosus*	bZIP transcription factor MafK	PIN25269.1	1407	468
10	Salicaceae	*Populus alba*	bZIP transcription factor 46-like	XP_034932357.1	1362	453

Note: CDS means coding sequence, ORF means open reading frame.

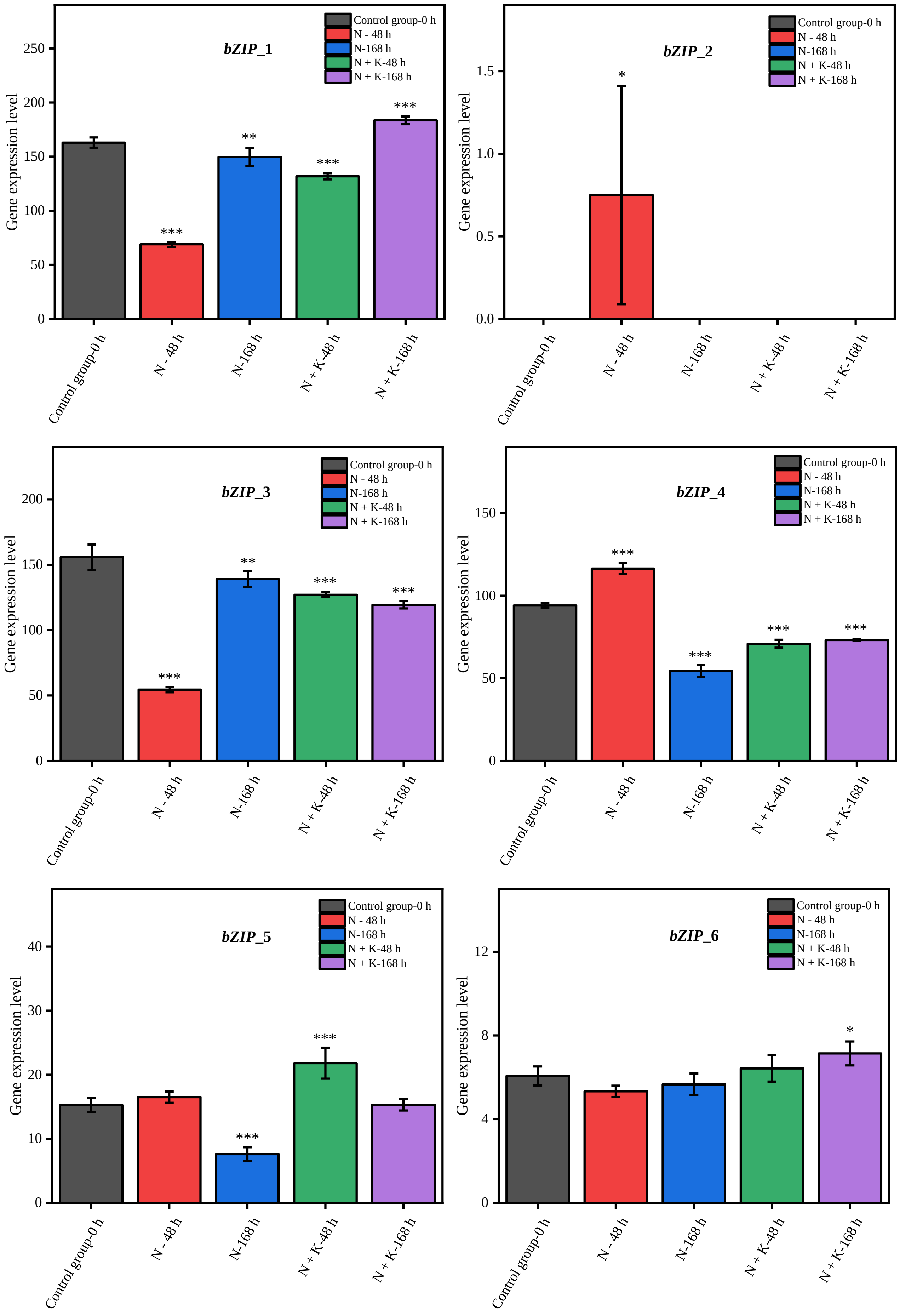
bZIP_1
bZIP_2
bZIP_3
bZIP_4
bZIP_5
bZIP_6
Control group-0 h
N - 48 h
N-168 h
N + K-48 h
N + K-168 h
Gene expression level
0
50
100
150
200
250
0.0
0.5
1.0
1.5
4
8
12
10
20
30
40
*
**
***

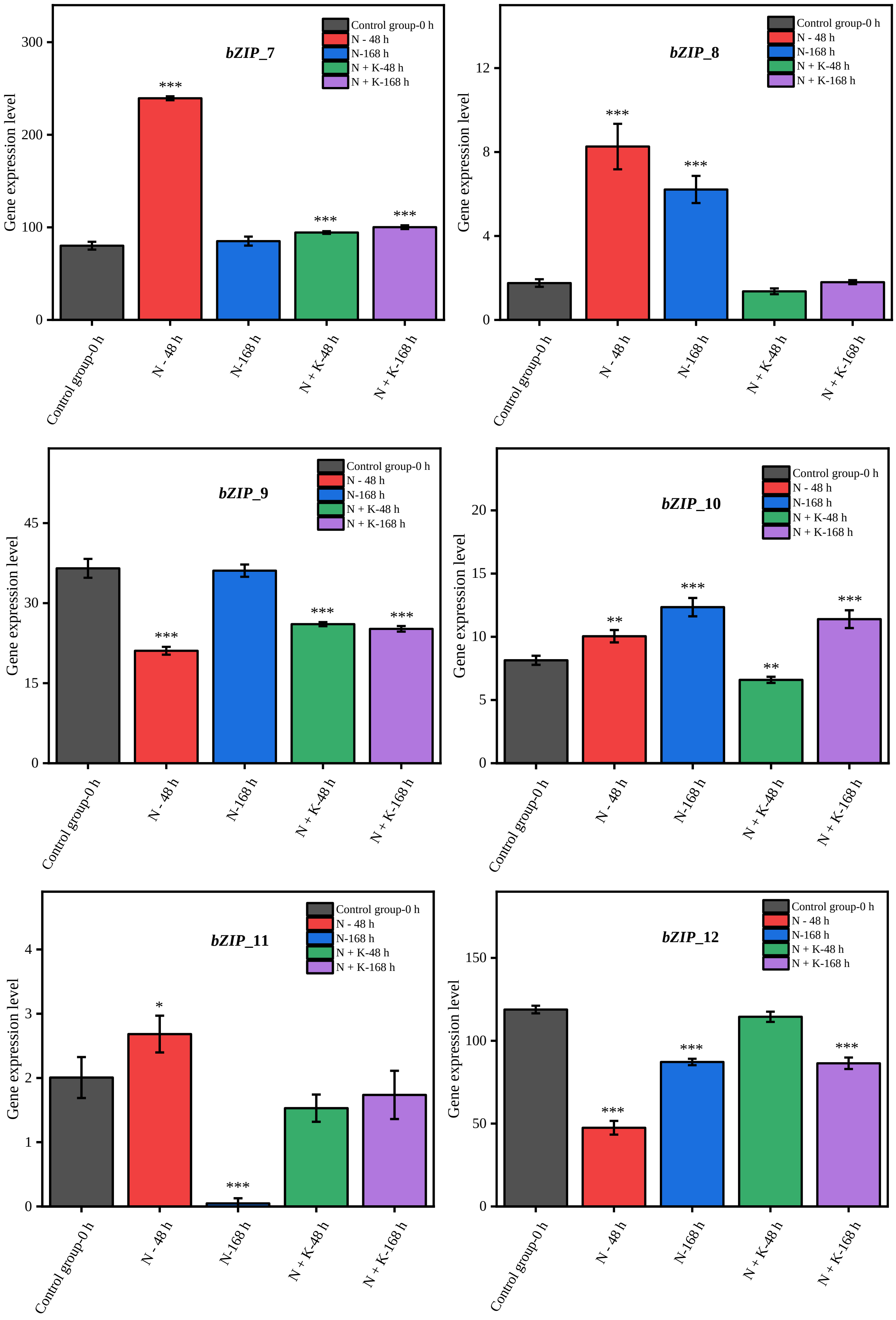
bZIP_7
Control group-0 h
N - 48 h
N-168 h
N + K-48 h
N + K-168 h
Gene expression level
0
100
200
300
***
***
***
Control group-0 h
N - 48 h
N-168 h
N + K-48 h
N + K-168 h
bZIP_8
Control group-0 h
N - 48 h
N-168 h
N + K-48 h
N + K-168 h
Gene expression level
0
4
8
12
***
***
Control group-0 h
N - 48 h
N-168 h
N + K-48 h
N + K-168 h
bZIP_9
Control group-0 h
N - 48 h
N-168 h
N + K-48 h
N + K-168 h
Gene expression level
0
15
30
45
***
***
***
Control group-0 h
N - 48 h
N-168 h
N + K-48 h
N + K-168 h
bZIP_10
Control group-0 h
N - 48 h
N-168 h
N + K-48 h
N + K-168 h
Gene expression level
0
5
10
15
20
**
***
**
***
Control group-0 h
N - 48 h
N-168 h
N + K-48 h
N + K-168 h
bZIP_11
Control group-0 h
N - 48 h
N-168 h
N + K-48 h
N + K-168 h
Gene expression level
0
1
2
3
4
*
***
Control group-0 h
N - 48 h
N-168 h
N + K-48 h
N + K-168 h
bZIP_12
Control group-0 h
N - 48 h
N-168 h
N + K-48 h
N + K-168 h
Gene expression level
0
50
100
150
***
***
***
Control group-0 h
N - 48 h
N-168 h
N + K-48 h
N + K-168 h

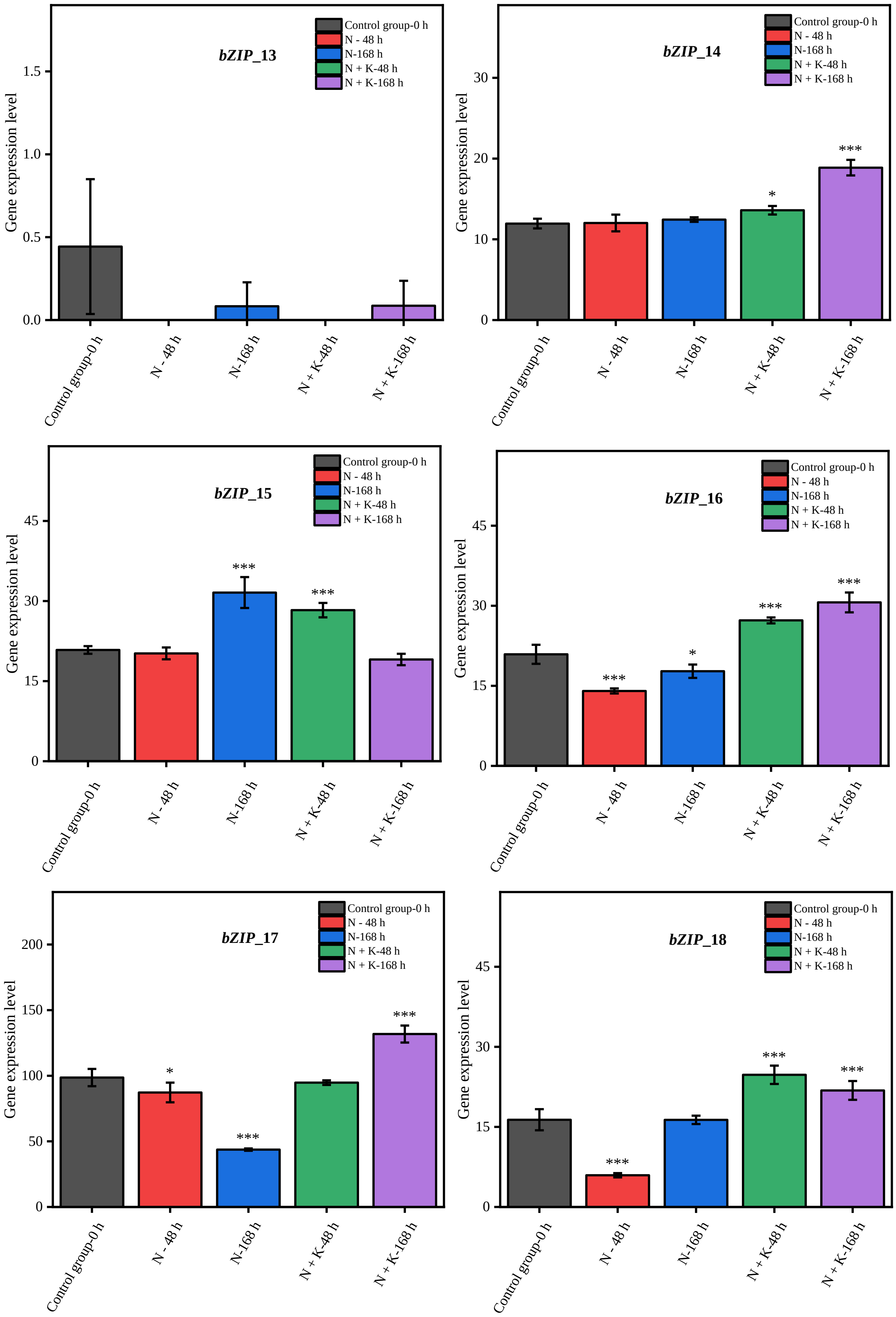

bZIP_13
bZIP_14
bZIP_15
bZIP_16
bZIP_17
bZIP_18
Gene expression level
Control group-0 h
N - 48 h
N-168 h
N + K-48 h
N + K-168 h
0.0
0.5
1.0
1.5
0
10
20
30
15
45
50
100
150
200
*
***

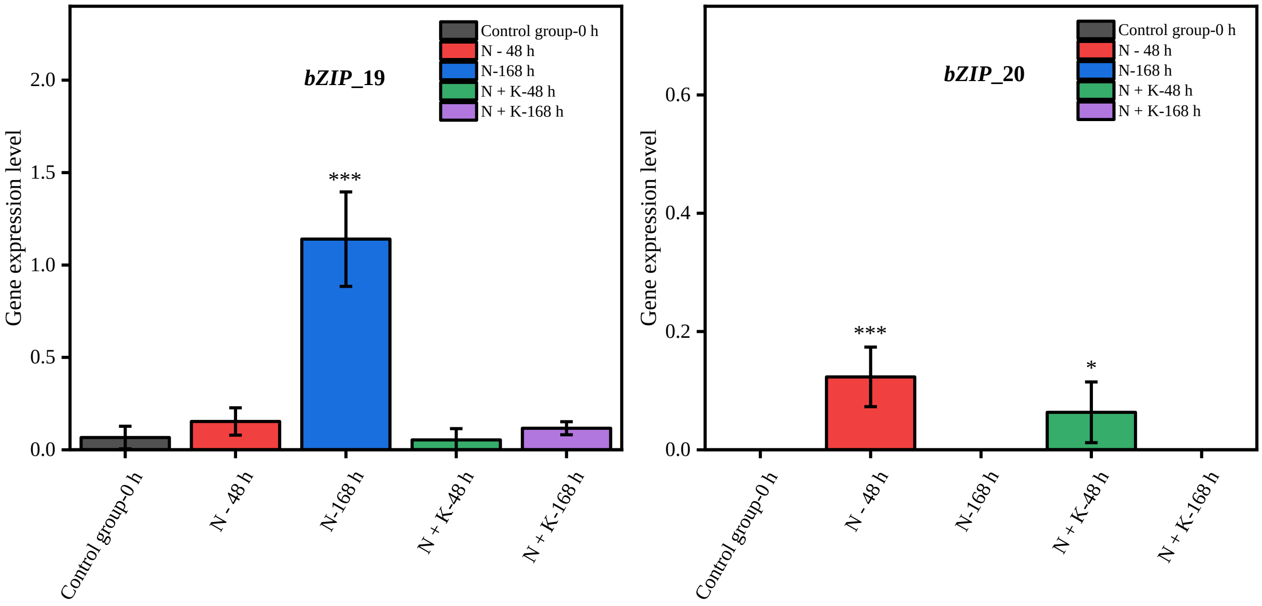

Supplementary Figure S1: Changes in the expression level of *bZIP* genes

(Changes in the expression levels of 20 *bZIP* genes in the roots of *T. ramosissima* under NaCl stress with exogenous K^+ applied for 48h and 168h. Note: $p \geq 0.05$ is not marked; $0.01 < p < 0.05$ is marked as *; $0.001 < p < 0.01$ is marked as **; $p \leq 0.001$ is marked as ***).
